# A Comparison of Immune Responses Exerted Following Syngeneic, Allogeneic, and Xenogeneic Transplantation of Mesenchymal Stem Cells into the Mouse Brain

**DOI:** 10.3390/ijms21093052

**Published:** 2020-04-26

**Authors:** Jung Won Hwang, Na Kyung Lee, Je Hoon Yang, Hyo Jin Son, Sa Ik Bang, Jong Wook Chang, Duk L. Na

**Affiliations:** 1 Department of Health Sciences and Technology, SAIHST, Sungkyunkwan University, 81 Irwon-ro, Gangnam-gu, Seoul 06351, Korea; 2 Stem Cell & Regenerative Medicine Institute, Samsung Medical Center, 81 Irwon-ro, Gangnam-gu, Seoul 06351, Korea; 3 School of Medicine, Sungkyunkwan University, 81 Irwon-ro, Gangnam-gu, Seoul 06351, Korea; 4 Samsung Alzheimer Research Center, Samsung Medical Center, 81 Irwon-ro, Gangnam-gu, Seoul 06351, Korea; 5 Laboratory Animal Research Center, Samsung Biomedical Research Institute, 81 Irwon-ro, Gangnam-gu, Seoul 06351, Korea; 6 Department of Neurology, Samsung Medical Center, Sungkyunkwan University School of Medicine, 81 Irwon-ro, Gangnam-gu, Seoul 06351, Korea; 7 Department of Plastic Surgery, Samsung Medical Center, Sungkyunkwan University School of Medicine, 81 Irwon-ro, Gangnam-gu, Seoul 06351, Korea; 8 R&D Center, ENCell Co. Ltd., Seoul 06072, Korea; 9 Neuroscience Center, Samsung Medical Center, 81 Irwon-ro, Gangnam-gu, Seoul 06072, Korea

**Keywords:** mesenchymal stem cell, immunogenic, syngeneic, allogeneic, xenogeneic

## Abstract

Due to their multifactorial aspects, mesenchymal stem cells (MSCs) have been widely established as an attractive and potential candidate for the treatment of a multitude of diseases. A substantial number of studies advocate that MSCs are poorly immunogenic. In several studies, however, immune responses were observed following injections of xenogeneic donor MSCs. In this study, the aim was to examine differences in immune responses exerted based on transplantations of xenogeneic, syngeneic, and allogeneic MSCs in the wild-type mouse brain. Xenogeneic, allogeneic, and syngeneic MSCs were intracerebrally injected into C57BL/6 mice. Mice were sacrificed one week following transplantation. Based on immunohistochemical (IHC) analysis, leukocytes and neutrophils were expressed at the injection sites in the following order (highest to lowest) xenogeneic, allogeneic, and syngeneic. In contrast, microglia and macrophages were expressed in the following order (highest to lowest): syngeneic, allogeneic, and xenogeneic. Residual human MSCs in the mouse brain were barely detected after seven days. Although the discrepancy between leukocytes versus macrophages/microglia infiltration should be resolved, our results overall argue against the previous notions that MSCs are poorly immunogenic and that modulation of immune responses is a prerequisite for preclinical and clinical studies in MSC therapy of central nervous system diseases.

## 1. Introduction

Mesenchymal stem cells (MSCs) can be defined as multipotent stem cells which can be readily isolated from many adult tissues such as bone marrow, umbilical cord, adipose, and Wharton’s jelly [1]. Once isolated from adult tissues, they can be cultured and expanded in vitro relatively easily for cell therapy [2]. MSCs possess self-renewing abilities and abilities for multilineage differentiation [3]. Thus, MSCs have demonstrated promising potential in a wide range of diseases, including neurodegenerative disorders such as Alzheimer’s disease (AD) [4,5].

Currently, there is no disease-modifying treatment available for AD. Theoretically, neural stem cells (NSCs) rather than MSCs appear to be more plausible for AD stem cell treatment since NSCs can differentiate into neurons and glial cells, repairing damaged tissue. However, recent stem cell research for AD has focused more on the therapeutic potential of multipotent MSCs rather than NSCs for the following reasons. First, MSCs are known for their short persistence [6] and limited differentiation when transplanted in vivo. Their limited differentiation abilities in vivo are a disadvantage from a regenerative medicine point of view. However, when considering the safety issues raised by the Food and Drug Administration (FDA), their short persistence serves an advantageous purpose. In this limited amount of time, it is reported that via a “hit-and-run” mechanism, MSCs are able to mediate therapeutic effects upon the host microenvironment [7,8]. Second, MSCs seem to increase therapeutic effects by their paracrine activities [9,10]. Previous studies suggested that the paracrine activity of MSCs in the host environment is effective in treating pathological symptoms of AD [10]. The myriad of paracrine factors secreted by MSCs increases the chances of targeting the multifaceted aspects of AD [5]. On the other hand, the paracrine activities of NSCs are yet to be elucidated. Third, unlike NSCs, MSCs are widely accepted to be poorly immunogenic. MSCs have attracted much attention due to their immunomodulatory capabilities, as recent findings suggest they are capable of influencing both innate and adaptive immunity [11,12,13]. Although the precise mechanism of immunomodulation by MSCs warrants further research, previous studies have reported on the immunosuppressive abilities of MSCs in a rodent model of graft-versus-host disease (GvHD) [14].

Contrary to the widely accepted belief that MSCs are poorly immunogenic, an increasing number of studies have highlighted the immunogenic properties of MSCs [15,16]. Interestingly, this immune reactivity seems to be not only generated from xenogeneic [17,18] but also from allogeneic MSC transplants [19,20]. Such results are surprising in that MSCs have been widely accepted to evade immune surveillance since they express low levels of MHC class I but do not express MHC class II [21]. Whether the proposed immune reactivity has an association with the low survival rate of the engrafted MSCs is questionable and has not been investigated extensively.

This study aims to investigate whether transplanting MSCs of varying origins (syngeneic vs. allogeneic vs. xenogeneic) into the brain parenchyma of wild-type mice generates different or similar immune responses. Originally proposed by Medawar, the central nervous system (CNS) has generally been regarded as an immune-privileged site [22]. Many groups have performed studies that involve xenogeneic intra-CNS grafts in a wide range of animal models [23,24,25,26]. Very few studies, however, have investigated whether immune responses will be exerted when these poorly immunogenic MSCs are stereotactically injected into the mouse brain parenchyma and whether the degree of the response will differ as a function of original MSC source. In concordance with recent findings, we hypothesized that MSCs would create the highest level of immunological response under xenogeneic experimental conditions and the lowest immune response under the syngeneic experimental condition. These results are expected to facilitate the successful use of MSCs for the treatment of neurodegenerative disorders including Alzheimer’s disease (AD).

## 2. Results

### 2.1. Confirmation of In Vitro Mesenchymal Trilineage Differentiation and Expression of Surface Antigens

**Figure 1:**
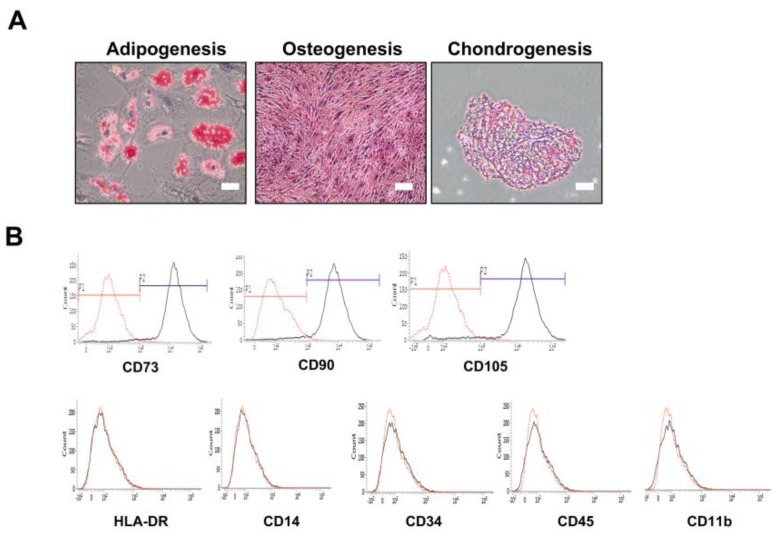
Validating the stemness of human adipose MSCs. (**A**) Adipogenic, osteogenic, and chondrogenic differentiating abilities of human adipose MSCs were validated by via Oil-red O (adipogeneis), Alizarin Red (osteogenesis), and Safranin O (chondrogeneis) staining, respectively. (**B**) Flourescence activated cell sorting (FACS) analysis of human adipose MSCs using positive (CD73, CD90, CD105) and negative (HLA-DR, CD14, CD34, CD45, and CD11b) markers. Scale bars = 200 µm.

The phenotypes of human adipose MSC (Figure 1) and ICR-derived MSCs (Supplementary Materials Figure S1) were characterized by referring to previous publications [27]. Human adipose MSCs were tested for their abilities to differentiate into adipocytes, osteoblasts, and chondrocytes (Figure 1A). They were responsive to adipocyte-inducing medium, showing positive results as red lipid vacuoles after Oil red O staining. Osteogenic response was also visible when incubated under osteogenic medium, with the MSCs exhibiting calcium deposits in the layers through Alizarin red staining [28]. A distinctive 3D pellet mass was formed following chondrogenic differentiation. Histological analysis after staining the pellet sections with Safranin O showed positive staining of the sulfated proteoglycan matrix and substantial lacunae full of type II collagen. Furthermore, human adipose MSCs possessed typical profiles of both positive and negative MSC surface markers. They expressed CD73, CD90, and CD105 but lacked expressions of HLA-DR, CD14, CD34, CD45, and CD11b (Figure 1B). Immunofluorescence imaging of ICR-mouse derived adipose MSCs showed positive stains of anti-FABP4, anti-Osteopontin, and anti-Collagen type II, defining their ability to differentiate into adipogenic, osteogenic, and chondrogenic lineages, respectively (Supplementary Materials Figure S1).

### 2.2. Successful Intra-Parenchymal Transplantation of Characterized MSCs

**Figure 2:**
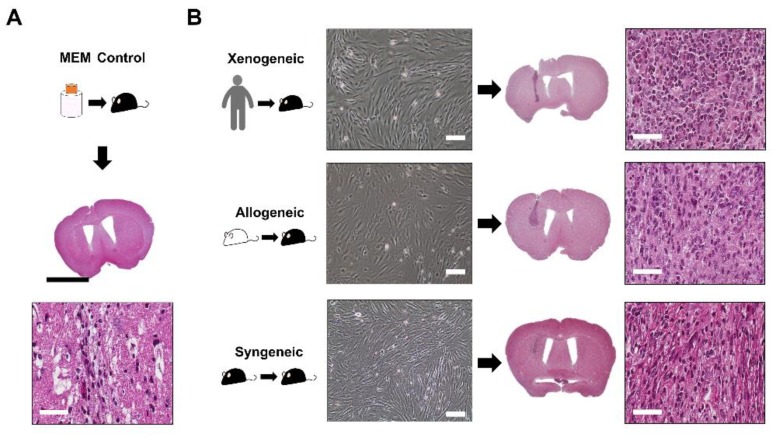
Transplantation of MSCs in the mouse caudate putamen confirmed via H&E staining. (**A**) The injection site was identified following MEM injection. CD45-positive leukocytes were barely observed at the injection site. (**B**) MSCs from all three groups (xenogeneic, allogeneic, and syngeneic) shared typical fibroblastic and proliferative profiles in vitro. Images were taken when cells reached 80%–90% confluency. H&E staining of mice brain tissues transplanted with MSCs from three different sources (xenogeneic, allogeneic, and syngeneic). The injection tract was clearly visible in the caudate putamen of mice (intense dark purple). Scale bars = whole brain: 2 mm, H&E (magnified): 50 µm, and in vitro cell images: 100 µm.

The MSCs from all three sources were cultured and grown in a monolayer. They all shared the fibroblast-like morphology after adhering to the culture plate, exhibiting that they possess proliferative capabilities (Figure 2). Following transplantation of the MSCs into the mouse parenchyma, the injection sites were first identified through Hematoxylin and Eosin (H&E) staining. When injected with Minimum Essential Medium (MEM), the injection site was identified as a vertical streak (Figure 2B). Compared to the syngeneic group, the injection sites appeared darker for the xenogeneic and allogeneic groups (Figure 2B). When magnified, pyknotic cells were identified at the injection site, but it was not possible to discern whether these pyknotic cells originated from the transplanted xenogeneic MSCs or the immune cells of the recipient.

### 2.3. Infiltration of CD45 Positive Leukocytes Was Highest in the Xenogeneic and Lowest in the Syngeneic Group

**Figure 3:**
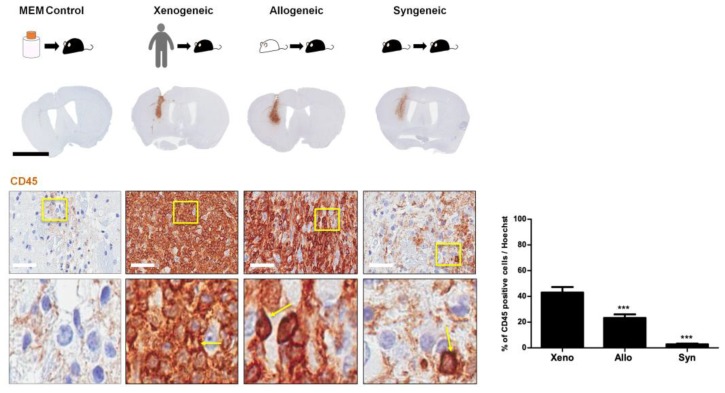
High Infiltration of CD45-positive leukocytes was evident at the site of MSC injection in the xenogeneic group. CD45-positive leukocytes were barely observed in the MEM-injected group. Infiltration of CD45-positive leukocytes (positive signals indicated in solid yellow arrows) was discernible at the site of injection. The highest infiltration was observed in the xenogeneic (xeno) group and the lowest in the syngeneic (syn) group. A statistically significant difference was observed when comparing CD45 expression levels between the allogeneic (allo) and xenogeneic (xeno) groups. Statistical significance was defined as *** *p* < 0.001 vs. xeno (xenogeneic); mean ± S.E.M. (A) Scale bar: whole brain: 2 mm, magnified image: 50 µm.

Immunological reaction following transplantation was assessed via CD45 staining (Figure 3). Strong immunological reactions were displayed in C57BL/6 mice transplanted with xenogeneic and allogeneic MSCs (dark brown color is indicative of severe infiltration of immune cells). A high infiltration of small, circular cells with a round nucleus (indicated by solid yellow arrows) was observed at the injection site (Figure 3). Positive CD45 leukocytes were barely identified at the injection site of the MEM control group. The percentages (mean ± S.E.M.) of CD45-positive leukocytes at the injection site were as follows: xenogeneic (43.1 ± 4.2%), allogeneic (23.3 ± 2.8%), and syngeneic (2.8 ± 0.6%) (Figure 3). The xenogeneic group showed the highest while the syngeneic group showed the lowest leukocyte infiltration (~15-fold difference). The allogeneic group did show reduced leukocyte infiltration in comparison to the xenogeneic group (~1.8-fold difference). As expected, STEM121-positive cells were not identified in the allogeneic and syngeneic groups (Supplementary Materials Figure S2).

### 2.4. Recruitment of Other Inflammatory and Immune Cells to the Injection Site Was Identified

**Figure 4:**
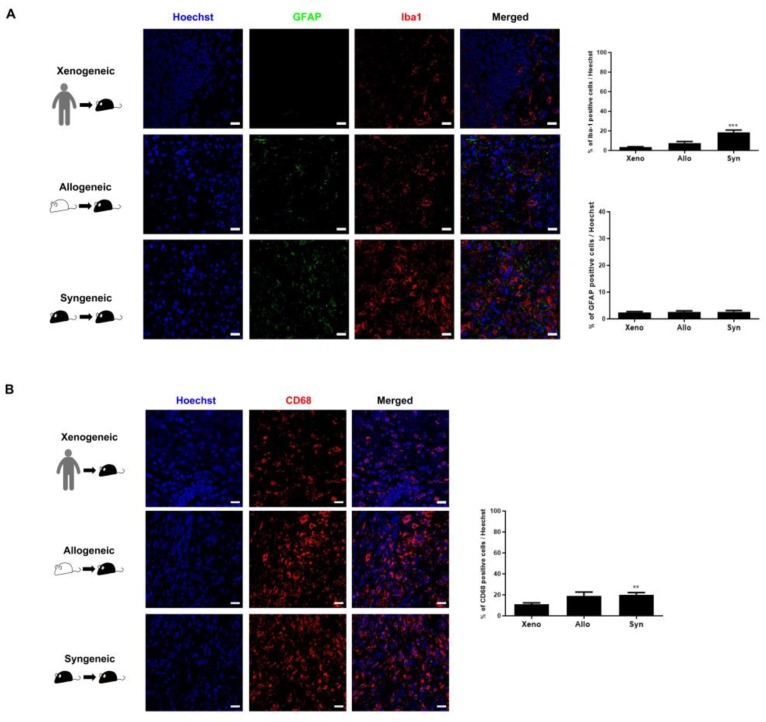
Highest Iba-1 and CD68 expression levels were identified in the syngeneic group. (**A**) The expression of GFAP-positive astrocytes was extremely low compared to that of Iba-1-positive microglia for all three groups. The highest expression of Iba-1-positive microglia was discernible in the syngeneic (syn) group and the lowest was identified in the xenogeneic (xeno) group. (**B**) Lowest number of CD68-positive macrophages occurred in the xenogeneic (xeno) group, whereas the highest number of CD68-positive macrophages occurred in the syngeneic (syn) group. Statistical significance was defined as ** *p* < 0.01, *** *p* < 0.001 vs. xenogeneic (xeno); mean ± S.E.M. Scale bars = 20 µm.

Other than the infiltration of CD45-positive leukocytes, the presence and proliferation of inflammatory cells such as microglia (anti-Iba-1), astrocytes (anti-GFAP), macrophages (anti-CD68), and other types of immune cells such as neutrophils (anti-neutrophil) at the injection sites of the three groups (xenogeneic, allogeneic, and syngeneic) were further assessed via IHC staining. Co-immunostaining was performed using anti-Iba1 and anti-GFAP (Figure 4A). Regarding the expressions of inflammatory cells (microglia, astroglia, and macrophages), first, the syngeneic group showed the highest expression levels of Iba-1-positive microglia (18.7 ± 2.2%) at the injection site, followed by the allogeneic (7.6 ± 1.5%), and lastly the xenogeneic (3.6 ± 0.4%) group (Figure 4A). Second, the expression levels of GFAP-positive astrocytes were overall relatively low for all three groups. A significant difference did not exist among the groups (xenogeneic; 2.5 ± 0.4%, allogeneic; 2.5 ± 0.5%, and syngeneic; 2.7 ± 0.6%) (Figure 4A). Third, within the CD45-positive leukocyte population, monocyte-derived macrophages may be involved in MSC clearance. Thus, we used the anti-CD68 antibody to observe the presence of macrophages at the site of MSC engraftment. A relatively high number of macrophages were present at the site of cell engraftment. Overall, the level of CD68 expression was highest in the syngeneic (20.2 ± 1.9%), followed by the allogeneic (18.8 ± 3.8%), and the lowest in the xenogeneic group (10.8± 1.6%) (Figure 4B).

**Figure 5:**
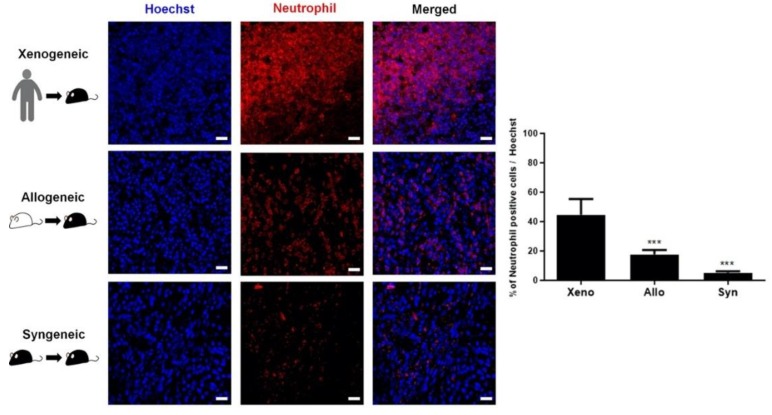
Extremely high number of neutrophils was identified at the injection site of the xenogeneic group. A massive recruitment of neutrophils was discernible at the injection site of the xenogeneic (xeno) group. A striking difference in neutrophil proliferation was evident when comparing the xenogeneic to the allogeneic (allo) and syngeneic (syn) groups. Statistical significance was defined as *** *p* <0.001 vs. xenogeneic (xeno); mean ± S.E.M. Scale bars = 20 µm.

Since neutrophils play an important role in innate immunity and are one of the major types of leukocytes (immune cells) that are abundantly present in humans [29], the presence and proliferation of neutrophils at the injection sites of the three groups were assessed further. IHC results acquired using the anti-neutrophil antibody were similar to those of CD45: The percentage of neutrophils was strikingly higher compared to that identified in the xenogeneic (44.7 ± 10.6%) group, which was followed by the allogeneic (17.7 ± 3.0%) and the syngeneic (5.2 ± 1.0%) groups (Figure 5).

### 2.5. CD8 T Cell Expression Was Relatively Low for All Three Groups

**Figure 6:**
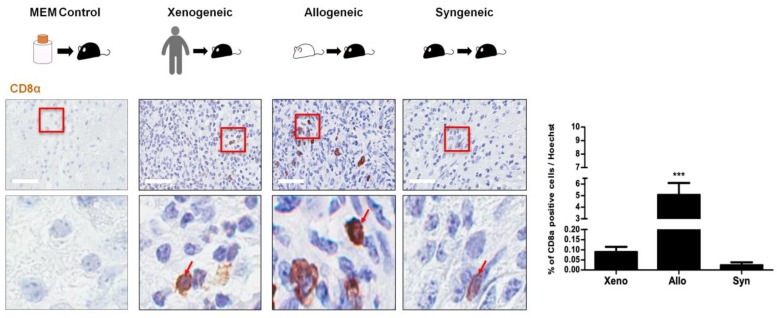
Relatively low expressions of CD8 T cells were identified at the injection sites in all three groups. Very few CD8 T cells (indicated as solid red arrows) were identified at the injection site, including the MEM-injected group. Compared to the xenogeneic (xeno) group, the highest number of CD8 T cells was observed in the allogeneic (allo) while the lowest number was observed in the syngeneic (syn) group. Red solid frames indicated magnified area of CD8 T cells. Statistical significance was defined as *** *p* < 0.001 vs. xenogeneic (xeno); mean ± S.E.M. Scale bars = 50 µm.

In addition to assessing the expressions of CD45-positive leukocytes and various inflammatory/immune cells at the injection site, the expression of cytotoxic T cells was also evaluated. Overall, the expressions of CD8 T cells were markedly reduced in all groups (Figure 5). Again, positive CD8 T cells were barely identified in the MEM-injected group (Figure 6). Small, round, oval-shaped CD8-positive T cells (solid red arrows) were identified in the vicinity of the injection sites of the xenogeneic, allogeneic, and syngeneic groups (Figure 6). The percentages (mean ± S.E.M.) of CD8 T cells at the injection site were as follows: xenogeneic (0.09 ± 0.03%), allogeneic (5.1 ± 1.0%), and syngeneic (0.02 ± 0.01%) (Figure 6). For the xenogeneic group, the proliferation of CD8 T cells was profoundly reduced compared to the number of CD45-positive leukocytes that was detected at the injection site. Unexpectedly, the allogeneic group had the highest number of CD8 T cells at the site of injection. T cell infiltration can influence the extravasation of immunoglobulin(IgG) into the brain [30]. Thus, the extent of mouse IgG leakage into the brain parenchyma was evaluated across the three groups using HRP-labeled anti-mouse secondary antibody. IgG staining was most intense in the xenogeneic group (5.6 ± 2.2%), and a slightly lower level of IgG was detected in the allogeneic group (5.3 ± 1.4%). At seven days post MSC injection, there was hardly any leakage of IgG detected in the syngeneic group (0.5 ± 0.2%) (Supplementary Materials Figure S3).

### 2.6. Xenogeneic MSC Persistence Was Significantly Reduced After One Week

**Figure 7:**
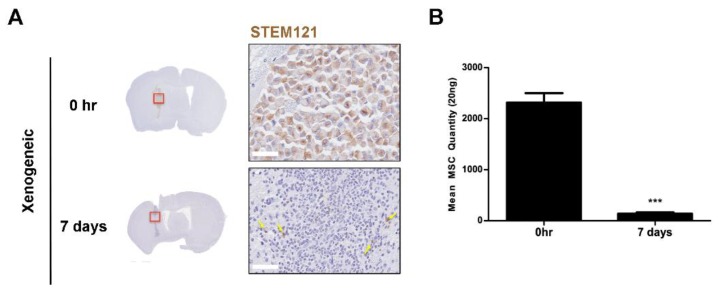
Persistence of human MSCs in the xenogeneic MSC group. (**A**) Based on immunohistochemical staining of anti-human STEM 121, barely any viable human MSCs were detected around the injection site seven days after injection. Red frames indicated magnified area of the injection sites and STEM 121-positive cells are indicated by solid yellow arrows. (B) Alu PCR quantification of remaining human adipose MSCs at 0 h and seven days post transplantation is shown on the right. Very few MSCs are present by seven days after MSC injection. Statistical significance was defined as *** *p* < 0.001 vs. 0 h; mean ± S.E.M. Scale bars: whole brain = 2 mm, magnified image: 50 µm.

The persistence of human adipose MSCs seven days after injection was examined by IHC (STEM121) and Alu PCR (Figure 7). While STEM121-postive cells were identified at the injection site of the 0-hr group, the presence of persisting human cells (solid yellow arrows) was barely discernible at seven days (Figure 7A). It was difficult to quantitate the number of STEM121-positive cells in the mouse parenchyma since so few were observed, and non-specific signals were also present at the injection site. Thus, a separate experiment was performed where the brain hemisphere (site where the injection was performed) was completely homogenized and the gDNA was extracted to perform PCR. Compared to the 0-hr group, a striking drop in the amount of persisting human MSCs (~17-fold difference) was detected in the seven-day group (Figure 7B). Human MSCs were barely present at seven days, which corroborated the histological results. 

## 3. Discussion

To the best of our knowledge, this is the first study to evaluate the effects of immune responses generated after transplanting adipose MSCs from varying origins (xenogeneic, allogeneic, and syngeneic) into the parenchyma of C57BL/6 wild-type mice. To prevent possible differences that may arise if MSCs from different sources are used, all groups were transplanted with MSCs isolated from the adipose tissue. If only pertaining to xenogeneic and allogeneic MSCs, the results of the study provide supporting evidence that MSCs are not poorly immunogenic. As expected, the lowest CD45 leukocyte infiltration was observed in the syngeneic group while the highest was observed in the xenogeneic group. Compared to the xenogeneic group, the percentage of CD45-positive leukocytes was markedly reduced in the allogeneic group. Recently, it has been proposed that MHC-mismatched allogeneic MSCs are more susceptible to immune rejection than MHC-matched allogeneic MSCs [16]. Even though the CNS is considered an immune-privileged area [22,31], immune responses against the transplanted MSCs were not thwarted. Even under these non-inflammatory conditions, where the poorly immunogenic MSCs were injected into the parenchyma of normal, WT mice, immune responses were not limited, as exhibited in the CD45 expression levels of the xenogeneic and allogeneic groups. In accordance with our study, a similar observation was made in a recent study that involved transplantations of human, canine, and murine MSCs into the ventricles of C57BL6/N mice [18]. An infiltration of T lymphocytes was observed upon administration of xenogeneic MSCs while such responses were absent from syngeneic MSC transplantation. 

Other than CD45-positive leukocytes, we performed additional immunostaining using various markers to examine the different populations of other inflammatory/immune cells present at the injection sites of the three groups. The family of leukocytes consists of various types of cells including monocytes, lymphocytes, and neutrophils. It has been reported that neutrophils are the major type of leukocytes, making up 50%–70% of the leukocyte population [32]. As expected, similar trends were observed in our study when comparing the CD45 and neutrophil IHC results. While the three groups showed no striking differences in GFAP-positive astrocyte expression, a significant difference was observed in Iba-1-positive microglia expression. Contrary to our expectation, however, the syngeneic group showed the highest expression of Iba-1-positive microglia followed by the allogeneic group. We do not have a clear explanation as to why such a high fold difference (~5.2 fold) existed in the Iba-1 microglial expression levels between the xenogeneic and syngeneic groups. There have been studies in the past, though, that have observed microglial recruitment from syngeneic cell grafts. For instance, microglial cells have been reported to accumulate in and around the vicinity of syngeneic mesenchymal stem cell grafts seven days after transplantation into the central nervous system [33]. In another study, an increased number of microglia was observed in syngeneic and allogeneic neonatal neural retina grafts, 12 days after transplantation [34]. Expression levels, though, subsided by day 35. Thus, it is highly possible that if sacrificed at a later time point other than one week, microglia expression levels might not have been pronounced in the syngeneic group in our study. Given the high number of CD45-positive leukocytes observed in the xenogeneic group, it is again unclear as to why the level of macrophage and microglia expressions was low in the xenogeneic group. 

Other than neutrophils and microglia, our results show that CD68-positive macrophages were present at the site of MSC engraftment. Such observations have been made in the past where intravenously infused MSCs in mice were eventually cleared by monocytic cells [35], and infiltration of CD68 inflammatory cells gradually increased over time following transplantation of rat MSCs into the hearts of rats that have underwent myocardial infarction [36]. The CD68 results were again contrary to our expectations that CD68 macrophage expression would be high for the xenogeneic group. However, unlike the expression of Iba-1-positive microglia, only a ~1.9-fold change existed when comparing the CD68 macrophage expression levels between the xenogeneic and syngeneic groups. The mechanism underlying why immune/inflammatory processes differed among the three MSC groups (xenogeneic, allogeneic, and syngeneic) is yet to be elucidated.

Interestingly, the expression level of CD8 T cells at the injection site was much lower than that of the CD45 leukocyte population across the experimental groups. This could have occurred due to the activation of the innate immune cells. Possibly due to the prevailing dogma that MSCs possess immunomodulatory capabilities, very few groups have investigated the expressions of CD45 leukocytes and CD8 T cells following transplantation of human MSCs into the mouse brain. In the past, a group investigated how human leukocytes mediate the rejection of porcine skin grafts. The group suggested that xenograft rejection is not influenced by the presence of CD8 T cells [37]. Moreover, another group presented findings that T cell infiltration is limited at the site of mesenchymal cell grafts [38]. 

Unexpectedly while CD8 T cells were barely expressed in the xenogeneic and syngeneic groups, CD8 T cell expression in the allogeneic group was relatively high. Since MSCs express very low levels of MHC class I, it was expected that MSCs would evade the detection mechanisms of NK cells [39]. It has been reported that allogeneic MSCs can generate allo-immune responses [40,41] which is indicative of higher CD45 and CD8 T cell expression in the allogeneic groups. Compared to autologous MSCs, allogeneic MSCs are more prone to be lysed by CD8 T cells and NK cells [42]. This could possibly explain why more CD8 T cells were present in the allogeneic group in comparison with the xenogeneic and syngeneic groups. Furthermore, a recent study also claimed that CD8 T cells are cytotoxic to allogeneic adipose-derived MSCs due to granzyme B produced in the CD8 T cells [43]. It has been reported that T cell infiltration induces blood brain barrier (BBB) leakage and this subsequently increases the deposition of immunoglobulin (IgG) in the brain [30]. Interestingly, when comparing IgG leakage in the mouse brains of the three groups, the xenogeneic group showed the highest, the allogeneic group was slightly lower than the xenogeneic, and the syngeneic group showed the lowest percentage of IgG extravasation. Excluding the xenogeneic results, overall, these results were equivalent to the CD8 T cell results. However, as to why significant differences did not exist between the xenogeneic and allogeneic groups warrants further investigation. High IgG extravasation observed in the xenogeneic and allogeneic groups, though, can party explain why relatively extremely high recruitment of CD45-positive leukocytes and neutrophils was observed at the injection sites of the two respective groups.

Along with high infiltration of CD45-positive leukocytes, it was interesting to note the poor survival rate of xenogeneic MSCs at post seven days. The absence of STEM121-positive cells in the allogeneic and syngeneic groups provided evidence that there was no human cell contamination. It also signified that the homogenous nature of the murine MSCs was the sole reason underlying the presence of CD45-positive leukocytes in the respective groups. It was previously reported that rat MSCs administered via the intra-arterial route entrap in the precapillary levels [44] and that a majority of MSCs of human or rodent origin also die within 48 h following systemic administration [15]. However, very few have reported on the rapid death of MSCs engrafted into the mouse brain via focal delivery. It is not yet clear whether the infiltrating leukocytes compromise the viability of the transplanted xenogeneic MSCs. The underlying mechanism was not investigated in this current study. In terms of cell survival, one caveat of this study was that we did not assess cell survival after one week. However, since the number of persisting human MSCs in the mouse parenchyma was extremely low at post seven days, we assume that xenogeneic MSCs would barely be detectable if investigated at time points past seven days.

There are limitations in our study. First, we did not examine the persistence and survival of syngeneic and allogeneic mouse MSCs in the mouse parenchyma. Unlike human MSCs that can be identified and differentiated from the surrounding mouse tissue by using human-specific markers, it is difficult to differentiate transplanted cells where the origin of the cells is equivalent to the species of the transplant recipient. An indirect labeling method that involves the use of reporter genes could be used in future studies. Green fluorescent protein (GFP) and red fluorescent protein (RFP) are examples of reporter genes. An advantage of transfecting cells with GFP or RFP is that only viable cells would express the gene [45], and thus via in vivo imaging or IHC, signals would only be observed in the cells that have persisted and survived up to the respective time point. Whether or not the transfection procedure affects the stemness (cell surface antigen expression, trilineage differentiation) or the viability of the MSCs must be assessed prior to transplantation. Second, a lack of pericyte differentiation evaluation is another shortcoming of this study. As well as playing a vital role in vascular development and maturation, pericytes also have the ability to regulate lymphocytes and aid clearance of toxic cellular materials [46,47]. Not only this, but pericytes are also known to regulate the activity of other surrounding stem cells [48,49]. Given the recruitment of leukocytes such as microglia (Iba-1) and neutrophils around the brain parenchyma after MSC injection, it is highly likely that pericyte differentiation played a role in leukocyte regulation. In the future, the role of pericytes in leukocyte regulation after MSC transplantation into the immune-privileged brain should be further elucidated with few other additional pericyte markers such as PDGFRβ [50].

According to the results obtained from this study, autologous MSCs seem to be the optimal source for MSC therapy in CNS diseases, given that the syngeneic group showed the lowest infiltration of CD45-positive leukocytes and CD8 T cells. While autologous cell therapy is favored and is vastly used in clinical trials, a major drawback is that it is restricted in terms of immediate availability [51]. Allogeneic MSCs, on the other hand, are readily accessible in comparison to autologous MSCs. However, to what extent allogeneic MSCs invoke immune responses is still ambiguous and requires further investigation.

For autologous or allogeneic MSCs to be used in clinical trials, the safety and efficacy of these cells must be tested in animal models; thus, xeno-transplants must be performed. However, as shown in this present study as well as by other groups, xenogeneic MSC transplantation elicits immune responses, specifically infiltration of CD45-positive leukocytes. Such immune responses can subsequently not only affect the survival of MSCs but also the therapeutic efficacy of MSCs. When conducting preclinical as well as clinical trials in the future, researchers much take into consideration that animal experiments performed up to this point have mostly used xenogeneic MSCs and thus immune responses could have been exerted. The co-application of immunosuppressants with MSC administration can be a potential strategy to reduce adverse effects following transplantation [41,52]. Directly modifying MSCs by suppressing MHC class I or inducing the overexpression of immunosuppressive factors can also be another approach [15].

Another area that deserves further exploration is the field of epigenetics. Epigenetics involves the modification of gene expression without altering the DNA sequence [53,54]. Epigenetics plays a major role in human biology and pathology [55]. For example, for allergic (type 2) inflammation, T cells differentiate into Th2 cells which secrete a multitude of cytokines to the external environment [55]. Epigenetic factors regulate the differentiation of T cells and cytokines secreted from these cells [55]. Moreover, epigenetics plays a role in the differentiation of MSCs into adipocytes or osteocytes [56,57]. Emerging studies have suggested that epigenetic factors can influence the activity of natural killer cells [54] and others propose that epigenetic mechanisms can alter the activities of immune cells involved in allograft rejection [58]. To what extent epigenetic mechanisms might have influenced the results obtained in this study warrants further investigation. Future studies should also focus on bringing clarity to the downstream signaling mechanisms underlying the immune responses generated following MSC transplantation.

## 4. Materials and Methods

### 4.1. Ethical Statement

This study has been approved by the Institutional Animal Care and Use Committee (IACUC) of the Samsung Biomedical Research Institute (SBRI) at Samsung Medical Center (SMC). As an accredited facility of the Association for Assessment and Accreditation of Laboratory Animal Care International (AAALAC International), SBRI acts in accordance with the guidelines set by the Institute of Laboratory Animal Resources (ILAR).

### 4.2. Culture and Preparation of Mesenchymal Stem Cells

Murine mesenchymal stem cells isolated from adipose tissue of C57BL/6 mice were purchased (passage six at time of purchase) and cultured according to the manufacturer’s instructions (Cyagen, Santa Clara, CA, USA). C57BL/6 mouse adipose-derived mouse MSCs (syngeneic) were seeded in 75T flasks and grown in Mouse Mesenchymal Stem Cell basal medium (Cyagen, Santa Clara, CA, USA) supplemented with 10% FBS (Biowest, Riverside, MO, USA) and 0.5% penicillin/streptomycin (Gibco, Waltham, MA, USA) at 37 °C in a 5% CO_2_ incubator. Cells were grown until passage eight, with a change of culture medium every two days. When they reached 80%–90% confluency, cells were harvested with 0.25% Trypsin-EDTA (Gibco, Waltham, MA, USA). Cells were resuspended in phenol red-free Minimal essential alpha 1X medium (MEMα1x) (Gibco, Waltham, MA, USA) before injection. 

To obtain allogeneic MSCs, 4-month-old ICR mice were euthanized through CO_2_ inhalation, and adipose tissue located in the abdominal viscera was dissected and immediately stored in ice cold phosphate-buffered saline (PBS). After centrifugation at 1500 rpm for three minutes (min), harvested tissues were minced and then placed in MEMα1X medium containing 0.5 % collagenase I (Sigma, St Louis, MO, USA). The mixture was then placed in a shaker at 37 °C for one hour. Afterwards, collagenase activity was inactivated by adding MEMα1X containing 10% FBS and 0.5% gentamicin (Gibco, Waltham, MA, USA). The mixture was filtered using a 100 μm cell strainer (Falcon, Austin, TX, USA), and the filtered mixture was centrifuged at 1500 rpm for 3 min. The supernatant was aspirated, and the cell pellet was resuspended in MEMα1X containing 10% FBS and 0.5% gentamicin. The cell suspension was filtered again using a 40 μm cell strainer (Falcon, Austin, TX, USA) and centrifuged at 1500 rpm for 3 min. After aspirating the supernatant, the cell pellet was resuspended in MEMα1X containing 10% FBS and 0.5% gentamicin, and the cells were then seeded on a 60 mm plate dish. Cells were cultured for 2–3 days 37 °C in a 5% CO_2_ incubator before passaging. The cells were grown until passage five, and at 80%–90% confluency, cells were trypsinized and resuspended in phenol red-free MEM alpha medium (Gibco, Waltham, MA, USA) prior to injection.

Human mesenchymal stem cells were isolated from adipose tissues of healthy donors with informed consent (IRB no. 2016-07-102). MSCs were isolated using the same protocol used to isolate MSCs from the adipose tissue of ICR mice. The cells were grown until passage five, and at 80%–90% confluency, cells were trypsinized and resuspended in phenol red-free MEM alpha medium (Gibco, Waltham, MA, USA) prior to injection.

### 4.3. In Vitro MSC Characterization

For this study, MSCs were prepared in accordance with the criteria defined by the International Society for Cellular Therapy (ISCT) [59]. Flow cytometry and in vitro differentiation into adipogenic, osteogenic, and chondrogenic lineages were carried out to characterize human MSCs. Adipogenic differentiation of MSCs was performed by plating human MSCs in a 6-well plate (Falcon, Austin, TX, USA) at a density of 1 × 10^6^ cells/well. At around 80–90% confluency, differentiation was induced by incubating the cells with StemPro Adipocyte differentiation basal medium (Gibco, Waltham, MA, USA) for up to two weeks. The medium was changed every 2–3 days. After the two weeks, cells were washed in PBS and then fixed in 4% paraformaldehyde (PFA; Biosesang, Seongnam, Republic of Korea). Fixated cells were stained with Oil red O (Sigma, St Louis, MO, USA) according to previously reported methods [27] for lipid staining. For osteogenic differentiation, human MSCs were seeded at 1 × 10^6^ cells/well in a 6-well plate. At around 80%–90% confluency, differentiation was induced by incubating the cells with StemPro basal medium (Gibco, Waltham, MA, USA) for up to two weeks. The medium was changed twice a week. Fixated cells were stained with Alizarin red (Millipore, Temecula, CA, USA) for calcium staining. All stained slides were examined by using an inverted light microscope (Olympus, Tokyo, Japan))

To assess chondrogenic differentiation, 2 × 10^5^ cells were placed in a 15 mL tube and centrifuged for 5 min at room temperature (RT) to form a pellet. Pellets were grown at 37 °C in a 5% CO_2_ incubator with the lids loosened and induced into chondrocytes for up to four weeks. Within the four weeks, chondrogenic medium was replaced every two to three days. Chondrogenic medium consists of Dulbecco’s Modified Eagle Medium (DMEM; Gibco, Waltham, MA, USA) supplemented with 500 ng/mL BMP-6 (R&D Systems, Minneapolis, MN, USA), 10 ng/mL TGF-β3 (R&D Systems, Minneapolis, MN, USA), 50 mg/mL ITS (BD, USA), 50 ug/mL ascorbic acid (Sigma, St Louis, MO, USA), 0.6 ug/mL dexamethasone (Sigma, St Louis, MO, USA), 40 ug/mL L-proline (Sigma, St Louis, MO, USA), and 100 ug/mL sodium pyruvate (Sigma, St Louis, MO, USA). After the four weeks, pellets were frozen in OCT compound (Scigen, Waltham, MA, USA), and 5-µm sections were made using a cryostat, mounted on slides, and stained with Safranin O (Sigma, St Louis, MO, USA) for acidic proteoglycan staining. All stained slides were examined by using an inverted light microscope (Olympus, Tokyo, Japan). To carry out phenotype characterization of ICR mouse-derived adipose MSC (allogeneic), the cells were stained with anti-FABP4 (R&D, Minneapolis, MN, USA), anti-Collagen type-II (Chemicon, Temecula, CA, USA), and anti-Osteopontin (R&D, Minneapolis, MN, USA) to confirm their adipogenic, chondrogenic, and osteogenic differentiation abilities.

Flow cytometry was carried out with FACS Verse (BD Biosciences, San Jose, CA, USA). To prepare the samples, 5 × 10^5^ human MSCs suspended in PBS were incubated with FITC, PE, or APC-coupled primary antibodies for 30 min at room temperature (RT) in the dark. After the primary antibody incubation, cells were washed with PBS and fixed using 1% PFA. The antibodies include CD11b, CD14, CD34, CD45, HLA-DR, CD73, CD90, and CD105, (BD Biosciences, San Jose, CA, USA). IgG1 and IgG2a (BD Biosciences, San Jose, CA, USA) were used as mouse isotype controls. According to the manufacturer (Cyagen, Santa Clara, CA, USA), both C57BL/6 mouse adipose-derived and BALB/c mouse bone marrow-derived MSCs (BM-MSCs) showed ≥ 70% positivity for expression of cell surface antigens CD29, CD44, Sca1 and ≤ 5% expression of cell surface antigens CD31 and CD117. Both cells were also able to differentiate into adipocytes, osteocytes, and chondrocytes. To assess adipogenic, osteogenic, and chondrogenic differentiation, cells were stained with anti-mFABP4, anti-mOsteopontin (R&D, Minneapolis, MN, USA), and anti mCollagen type II (Chemicon, Temecula, CA, USA). All stained slides were analyzed under a confocal microscope (ZEISS, Oberkochen, Germany).

### 4.4. Experimental Animals

**Figure 8:**
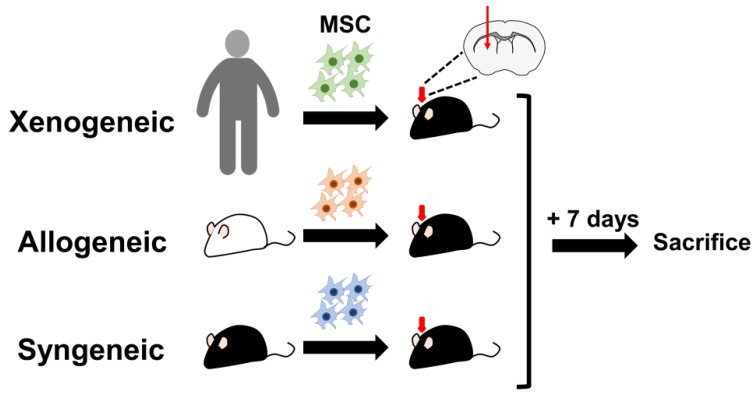
Schematic illustration of the experimental design. Xenogeneic (human MSCs), allogeneic MSCs (ICR MSCs), or syngeneic (C57BL/6 MSCs) were transplanted into the left caudate putamen of C57BL/6 mice. Mice were sacrificed one week after transplantation.

A total of 39 C57BL/6 (MEMα: *n* = 5, syngeneic: *n* = 5, allogeneic: *n* = 5, and xenogeneic: *n* = 14) and 10 ICR (four months old) female mice were used for this study. All the mice designated to the MEMα, syngeneic, and allogeneic groups were subjected to IHC staining following sacrifice. Out of the 17 mice that were used for the xenogeneic group, n = 6 was used for IHC staining and n = 8 was used for Alu qPCR (*n* = 3: 0 h, *n* = 5: post 1-week sacrifice). All 10 ICR mice were sacrificed to obtain adipose tissue. C57BL/6 and ICR mice were purchased from Orientbio (Seongnam, Republic of Korea). Mice were sacrificed one week following transplantation (Figure 8).

### 4.5. Transplantation of Mesenchymal Stem Cells into the Mouse Parenchyma

Following initial anesthetization with 5% isoflurane (Hana Pharmaceutical Co., Ltd.,Seoul, Republic of Korea), mice were placed on a rodent universal surgical stereotactic frame (Harvard apparatus, Holliston, MA, USA), and their heads were fastened using ear bars. During the injection, anesthesia was maintained at 1.5–2% isoflurane. A total of 2 × 10^5^ MSCs suspended in 5 μL of phenol red-free MEMα1X medium was injected into the left caudate putamen at the following coordinates: A/P: + 0.5 mm, M/L: + 1.7 mm, and D/V: −3.3 mm. Cells were injected at a rate of 0.5 µL per min by using a 25 µL Hamilton syringe (Hamilton Company, Reno, NV, USA). The syringe was withdrawn following a 5-min delay to minimize backflow. 

### 4.6. Histological Analysis and Quantification

At post seven days, mice were sacrificed through cardiac perfusion with PBS. The harvested brain tissue samples were fixed in 4% PFA for 24 h before being embedded in paraffin. Then, 4-µm-thick coronal sections were obtained using a microtome (Leica Biosystems, Wetzlar, Germany). After deparaffinization, heat-induced epitope retrieval was achieved by using sodium citrate buffer, pH 6.0 (Dako, Glostrup, Denmark). To block endogenous peroxidase activity, slides were incubated in peroxidase-blocking solution (Dako, Glostrup, Denmark) for 15 min. Additional blocking was done by treating slides with MOM blocking (Vector, USA) for 1 hr. Then, slides were treated with protein block serum-free ready-to-use for 20 min (Vector Laboratories, Burlingame, CA, USA) at RT. Afterwards, slides were treated with primary antibodies overnight at 4 °C. After performing multiple washings, slides were incubated in secondary antibody using HRP-labeled anti-mouse/rabbit/rat secondary antibody solutions (Dako, Glostrup, Denmark) for 30 min at RT. Slides then were treated with Dako liquid 3,3′-Diaminobenzidine (DAB) substrate chromogen system for 3 min and finally stained with hematoxylin (Dako, Glostrup, Denmark). Slides were mounted and scanned using a Scanscope AT scanner (Leica Biosystems, Wetzlar, Germany). The following primary antibodies were used: anti-rabbit CD8α (1:400; Cell Signaling, Danvers, MA, USA), anti-rat CD45 (1:200; Biolegend, San Diego, CA, USA), anti-mouse STEM 121 (1:500; Cellartis, Japan), anti-rabbit CD68 (1:200; Abcam, Cambridge, UK), anti-rabbit Iba-1 (1:250; Wako Chemicals, Osaka, Japan), anti-goat GFAP (1:1000; Abcam, Cambridge, UK), and anti-rat neutrophil (1:200; Abcam, Cambridge, UK). The following secondary antibodies were used for immunofluorescence staining: Alexa Fluor 488-conjugated goat anti-rat (1:400), Alexa Fluor 488-conjugated donkey anti-goat (1:400), Alexa Fluor 546-conjugated donkey anti-rabbit (1:400), Alexa Fluor 594-conjugated donkey anti-rat (1:400), and Alexa Fluor 594-conjugated goat anti-rabbit (1:200; Life Technologies, Carlsbad, CA, USA). IgG leakage was examined by treating slides with an HRP-labeled anti-mouse secondary antibody (Dako, Glostrup, Denmark) for 1 h at RT, excluding the primary antibody step. Slides were then visualized by using DAB. The % area of the DAB stain in the IgG stains was assessed by using the color deconvolution plugin in the ImageJ image analysis software (NIH, Bethesda, MD, USA). To quantify the number of CD45, CD68, Iba-1, GFAP, and neutrophil-positive cells at the site of injection, stained slides were scanned using the multispectral tissue imaging platform Vectra III (Perkin–Elmer, Waltham, MA, USA). From the whole scans (4×), regions of interests (ROIs) were drawn (669 × 500 μm) and ROIs were re-scanned at 20×. Quantification was carried out using the inForm^®^ software provided with the Vectra-III multispectral instrument.

### 4.7. Human Alu PCR Quantification of Residual Human MSCs in the Mouse Brain

A total of six C57BL/6 mice were injected with human MSCs (2 x 10^5^/ 5 μL) into the left caudate putamen according to procedures stated above. Three mice were sacrificed immediately after administration (used as a control), and three mice were sacrificed after seven days. The hemispheres of the harvested brain samples were separated and immediately frozen in liquid nitrogen. The Gentra Puregene Tissue Kit (Qiagen, Hilden, Germany) was used to extract genomic DNA from the brain tissue samples (left hemisphere only—the hemisphere where the injection was performed). Real-time polymerase chain reaction (PCR) was carried out using a mixture containing 20 ng of genomic DNA for each sample, SYBR Green Master Mix probe (Thermo Fisher Scientific, Waltham, MA, USA), and primers that targeted the human Alu element. Primers were designed by referring to a previously reported study [60]. The primer sequences were as follows: 5′-CAT GGT GAA ACC CCG TCT CTA-3′ (Alu Forward) and 5′–GCC TCA GCC TCC CGA GTA G-3′ (Alu Reverse). PCR conditions were carried out as previously reported [61]. DNA amplification was performed in 40 cycles using 95 °C for initial hold (10 min), 95 °C for denaturation (15 s), 68 °C for annealing (30 s), and 72 °C for extension (30 s). Genomic DNA was also extracted from a varying number of human MSCs (10^2^, 10^3^, 10^4^, 10^5,^ 10^6^), and the threshold cycle (C_T_) values were used to create a standard curve. The amount of residual human MSCs in the mouse brain samples was calculated by fitting the respective C_T_ values to the standard curve.

### 4.8. Statistical Analysis

All values are presented as the mean ± standard error of mean (S.E.M). One-way ANOVA or a *t*-test was used to assess significance, and a *p*-value ≤ 0.05 was considered statistically significant (GraphPad Prism, San Diego, CA, USA).

## 5. Conclusions

Taken collectively, these data indicate that xenogeneic and allogeneic MSCs elicit immune responses when transplanted into the parenchyma of wild-type mice. The additional measures that must be taken to reduce immune responses and whether the immune response affects the persistence of engrafted MSCs are pending questions that must be addressed in order to advance MSC therapy.

## Supplementary Materials

The following are available online at https://www.mdpi.com/1422-0067/21/9/3052/s1, Figure S1: Validating the stemness of ICR adipose MSCs, Figure S2: Human cell contamination is not identified from the allogeneic and syngeneic groups, and Figure S3: Higher IgG levels detected in xenogeneic and allogeneic groups.
