# Supplementary material for: A Comparison of Immune Responses Exerted Following Syngeneic, Allogeneic, and Xenogeneic Transplantation of Mesenchymal Stem Cells into the Mouse Brain
Source: Int J Mol Sci. 2020 Apr 26;21(9):3052. doi: 10.3390/ijms21093052 (PMC7246520; doi:10.3390/ijms21093052)
Supplement: Supplementary file 1 [file ijms-21-03052-s001.pdf]

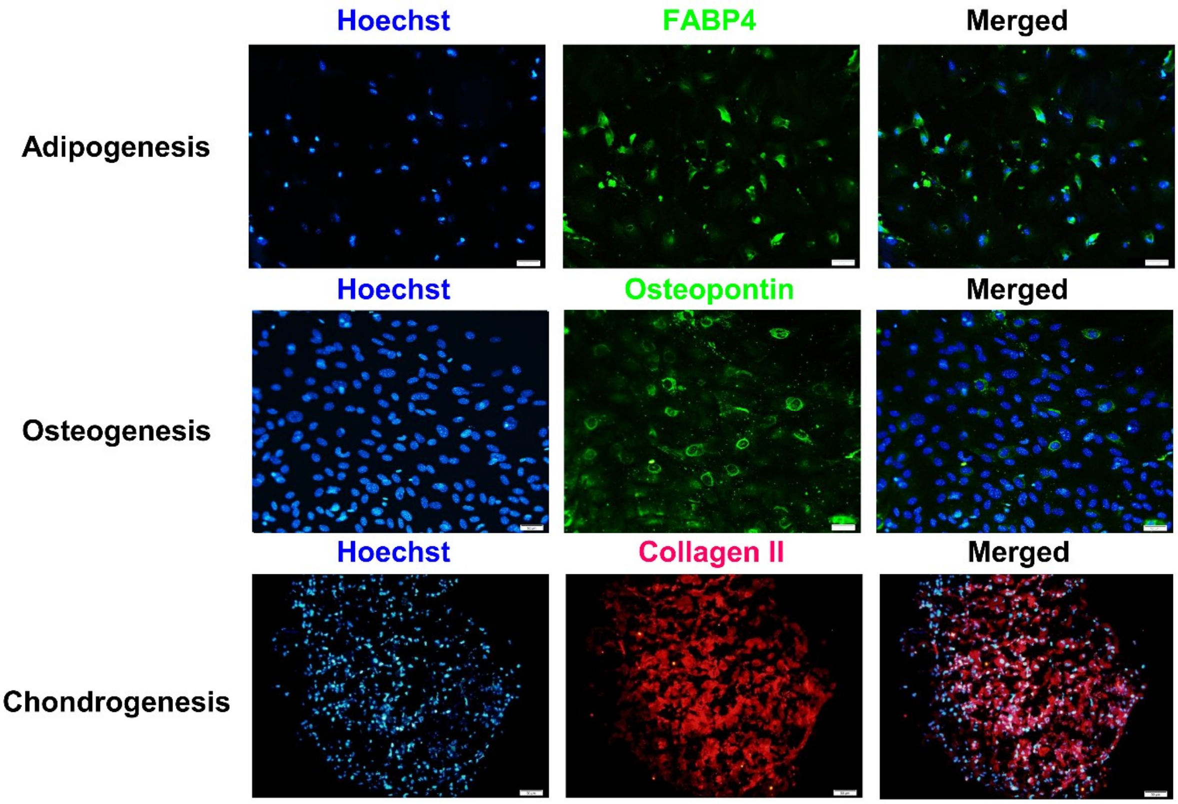

Figure S1. Validating the stemness of ICR adipose MSCs. ICR-derived adipose MSCs (allogeneic) staining with anti-mFABP4, anti-mOsteopontin, and anti-mCollagenII to validate their adipogenic, osteogenic, and chondrogenic lineages, respectively. Scale bars = 50 μm.

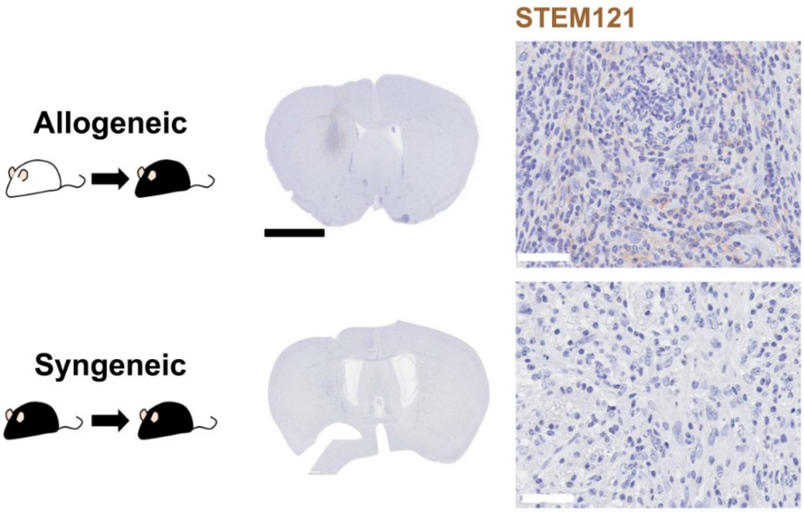

Figure S2. Human cell contamination is not identified from the allogeneic and syngeneic groups. No STEM121 positive cells are identified from the IHC stains of the allogeneic and syngeneic brain tissue sections. Scale bars: whole brain = 2 mm, magnified image: 50 μm.

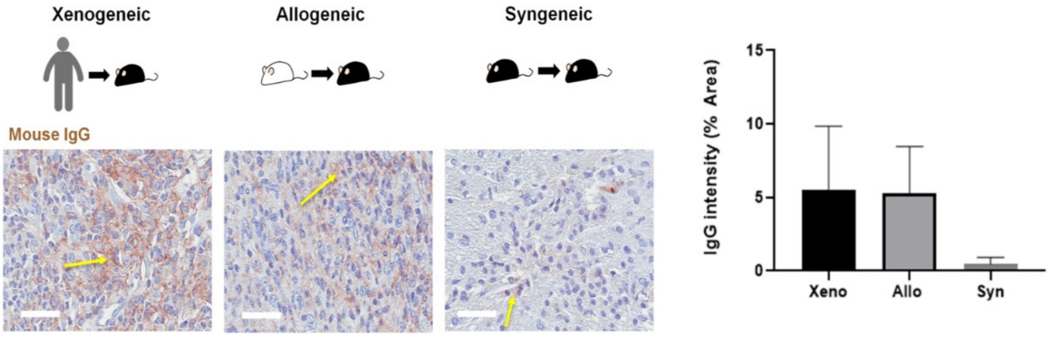

Figure S3. Higher IgG levels detected in xenogeneic and allogeneic groups. DAB staining with mouse IgG HRP shows highest level of IgG leakage in the xenogeneic (xeno) group and the lowest level of IgG extravasation in the syngeneic (syn) group (IgG leakage indicated by solid yellow arrows). Scale bar = 50μm.
